# Peas and Primroses

**Published:** 2005-03-15

**Authors:** Lynne S. Wilcox

Remember the monk and the peas? The story introduced school children to genetics long before the human genome made the evening news. The Augustinian monk Gregor Mendel conducted hundreds of experiments on the edible pea, crossing peas that were smooth or wrinkled, peas that grew tall or short, peas that had white or violet flowers. He found that the results of these crosses were predictable, and in 1865 he presented his findings to the Brunn Society for the Study of Natural Science in Moravia (1).

But there was little reaction to Mendel's report, and his next experiments took a different turn. Mendel sent his report to a famous botanist in Munich, Karl Wilhelm von Näli. The botanist replied, but his letter encouraged Mendel to examine the hawkweeds, a group of plants related to asters. In 1866, no one recognized that hawkweeds reproduced asexually and were not subject to Mendel's meticulously developed theories of hybridization. Mendel went through years of scientific failure, all the while continuing his hawkweed correspondence with Näli. By 1873 Mendel gave up his experiments and became abbot of the monastery. He died in 1884 without ever seeing his pea experiments vindicated (1).

Fiction writer Andrea Barrett, winner of the National Book Award and a MacArthur Fellowship (2), describes more about these experiments in her short story "The Behavior of the Hawkweeds," which sets the lives of 20^th^-century fictional characters, a geneticist and his wife and friends, against the background of these 19^th^-century historical figures (3). Barrett's fictional plot confirms that human relationships are more complex than they might first appear. Her interweaving of this story with Mendel's history demonstrates that the same is true for scientific progress. Mendel believed he had identified a new pattern, and indeed he had. But not all inherited traits followed his pattern, and scientists remained confused about hybridization for several more decades.

Modern public health professionals in the United States first addressed genetic diseases that followed single-gene (Mendelian) laws. Mass newborn testing in states began in the 1960s when it was recognized that phenylketonuria was a genetic disease that could be significantly ameliorated by diet if identified at birth. Over time more states began newborn testing, and more genetic diseases were added to the testing lists (4).

Today we recognize that specific gene patterns are associated with certain diseases, but the degree to which the first predict the second is more complex than Mendelian genetics can define. Diabetes, cancers, and cardiovascular diseases, for example, may result from several combinations of inheritance, environment, and other factors still unidentified. These interactions make it challenging to identify populations at highest risk. When does disease incidence, predictive value of genetic tests, and understanding of environmental influences become sufficient to consider population-based testing? When can public health professionals advise policy makers that testing would help prevent disease morbidity and mortality? When might alteration of environment be more efficient than mass testing? This issue of *Preventing Chronic Disease* explores these questions in the context of public health programs. Dr Muin Khoury, Director of the Office of Genomics and Disease Prevention at the Centers for Disease Control and Prevention, discusses several articles in this issue devoted to public health genomics (5). 

Mendel's laws of heredity were "rediscovered" in 1900 by several scientists acting independently. One of these was the Dutch botanist Hugo de Vries (6). He studied mutations in evening primroses and, in the process of explaining his results, learned of Mendel's pea research and theories. "Peas and primroses," Barrett writes, "primroses and peas, passing their traits serenely through generations" (3). Human genomics may be less serene than horticulture, but understanding how our traits are passed through generations is critical to the public's health.
